# Ilio-Hepatic Artery Bypass for Hypoplasia of the Celiac Axis and Its Branches with an Inferior Pancreaticoduodenal Artery Aneurysm

**DOI:** 10.3400/avd.cr.21-00059

**Published:** 2021-09-25

**Authors:** Masaru Nemoto, Tatsuki Watanabe, Yu Tadokoro, Yutaka Takayama, Junji Yamamoto

**Affiliations:** 1 Department of Surgery, Ibaraki Prefectural Central Hospital & Cancer Center, Kasama, Ibaraki, Japan; 2 Fujiyoshida Showa Clinic, Fujiyoshida, Yamanashi, Japan

**Keywords:** ilio-hepatic artery bypass, inferior pancreaticoduodenal artery aneurysm, hypoplasia

## Abstract

Hemorrhage due to a ruptured pancreaticoduodenal artery aneurysm is potentially fatal. We describe a case of a 51-year-old man, incidentally diagnosed with an inferior pancreaticoduodenal artery aneurysm associated with probable congenital hypoplasia of the celiac axis and its branches. Considering the rupture risk, we performed an ilio-hepatic artery bypass with an autologous vein graft and aneurysmorrhaphy. The postoperative course was uneventful. At the 24-month follow-up, the bypass was patent, with no aneurysm recurrence. The ilio-hepatic artery bypass is effective and preserves visceral blood flow. However, the iliac artery is susceptible to occlusive disease, and long-term follow-up is required.

## Introduction

Inferior pancreaticoduodenal artery (IPDA) aneurysms are uncommon visceral aneurysms, sometimes associated with celiac axis stenosis or occlusion.^1)^ The major concern for IPDA aneurysms is the risk of rupture and subsequent mortality. The size of the aneurysm does not seem to predict rupture; therefore, prompt endovascular or surgical procedures are necessary.^2)^ We describe an IPDA aneurysm caused by suspected congenital hypoplasia of the celiac axis, common hepatic artery, splenic artery, and left gastric artery, treated with an extra-anatomical bypass, an autologous vein graft, and aneurysmorrhaphy.

## Case Report

**Figure 1:**
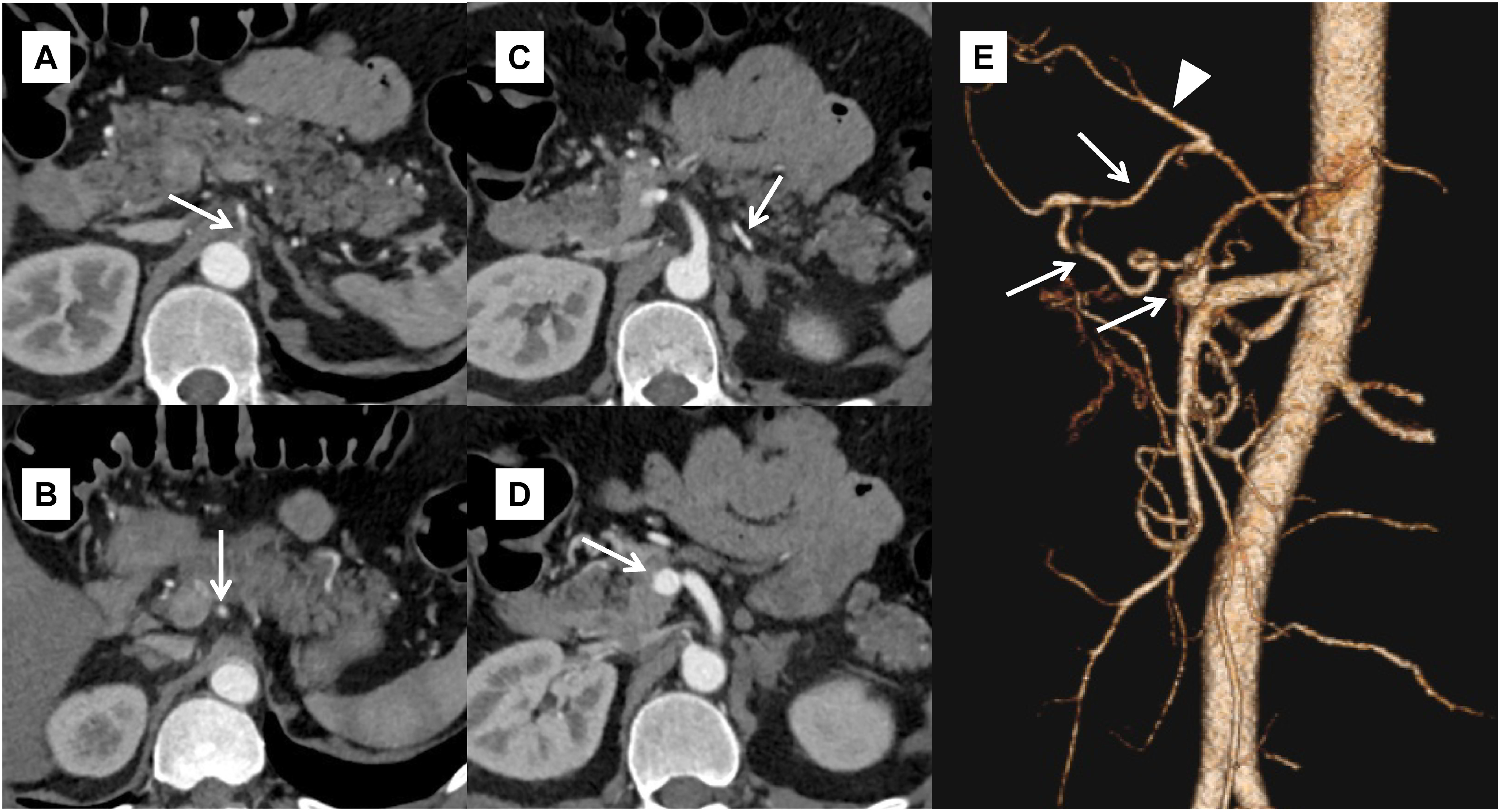
Fig. 1 Computed tomography (CT) reveals hypoplasia of the celiac axis (**A**, arrow), common hepatic artery (**B**, arrow), and splenic artery (**C**, arrow). The inferior pancreaticoduodenal artery (IPDA) aneurysm, 11 mm in size, branched from a dilated superior mesenteric artery, is close to the origin of the IPDA (**D**, arrow). Three-dimensional CT indicates the IPDA aneurysm with the development of pancreatic arcades (**E**, arrows), and the proper hepatic artery (**E**, arrowhead).

**Figure 2:**
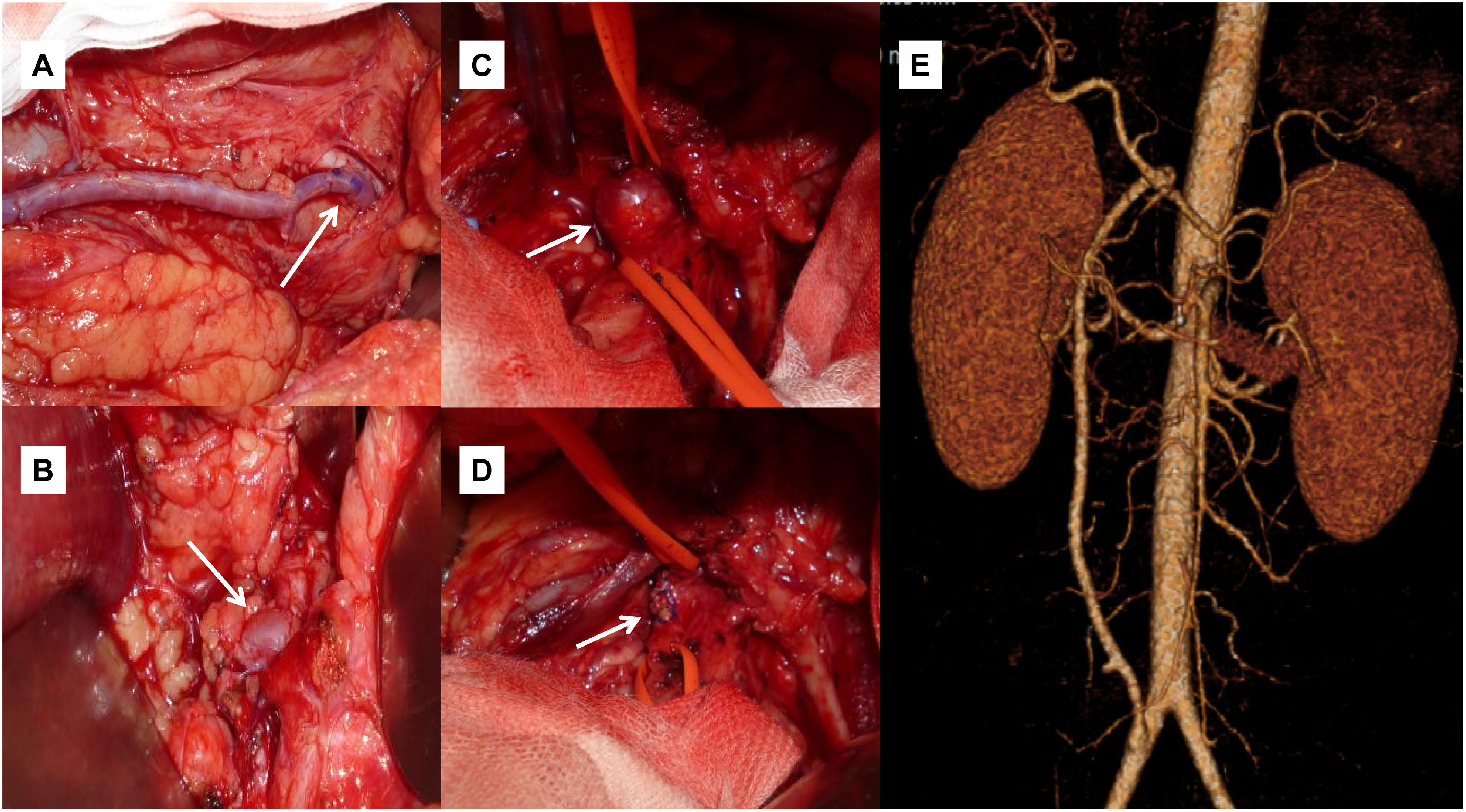
Fig. 2 The proximal (**A**, arrow) and distal (**B**, arrow) anastomoses of the ilio-hepatic artery bypass with an autologous vein graft through the right retroperitoneal space are indicated. The inferior pancreaticoduodenal artery aneurysm is detected (**C**, arrow), and an aneurysmorrhaphy is performed (**D**, arrow). Three-dimensional computed tomography indicates the patency of the graft and no recurrence of aneurysms (**E**).

A 51-year-old man with a duodenal ulcer and no history of alcohol intake or smoking presented to another hospital complaining of melena. He was referred to our hospital, where an emergency upper gastrointestinal endoscopy revealed a gastric ulcer at the lesser curvature of the pylorus. Contrast-enhanced computed tomography (CT) incidentally revealed the hypoplastic celiac axis, common hepatic artery, splenic artery, and left gastric artery, with an 11-mm IPDA saccular aneurysm branched from the dilated superior mesenteric artery (SMA) (**Fig. 1**). The aneurysm was near the SMA trunk. The proper hepatic artery was not hypoplastic; the other vessels showed no atherosclerotic changes. Ultrasonography revealed retrograde flow in the gastroduodenal artery (GDA). Considering the rupture risk of the saccular aneurysm, we planned a surgical bypass with aneurysm exclusion after his gastric ulcer improved. Through a midline incision, we selected the right common iliac artery as an inflow and bypassed it to the proper hepatic artery with a great saphenous vein graft through the right retroperitoneal space. Then we performed an aneurysmorrhaphy (**Figs. 2A**–**2D**). There was no intraoperative evidence of compression of the celiac axis with the median arcuate ligament. Postoperatively, the flow of the GDA became antegrade. The postoperative course was uneventful, and the patient was discharged on postoperative day 11. The pathological examination of the patient’s aneurysmal wall revealed atherosclerotic changes based on intimal fibrotic thickening and partial calcification. We did not prescribe antithrombotic drugs. A CT scan 24 months later revealed the patency of the graft and no recurrent aneurysms (**Fig. 2E**). The ethics committee of our institution approved this study, and the patient provided written informed consent for the publication of this case report.

## Discussion

IPDA aneurysms are uncommon and are associated with trauma, pancreatitis, infection, surgical procedures, and systemic vasculitis.^3)^ Celiac axis stenosis or occlusion, induced by atherosclerosis or median arcuate ligament syndrome, has also been reported due to increased retrograde flow to the pancreaticoduodenal arterial arcades from the SMA the formation of aneurysms.^4)^ The critical problem with these aneurysms is a rupture risk with a high mortality rate of up to 50%, reportedly not associated with the aneurysmal diameter, requiring prompt intervention.^2,5)^ In this case, the celiac axis, common hepatic artery, splenic artery, and left gastric artery were hypoplastic, despite no obvious risk factors for atherosclerosis and no relevant history, such as pancreatitis and infection or systemic vasculitis. Segmental arterial mediolysis was impossible based on the pathological findings; therefore, we suspected congenital arterial hypoplasia. Despite the causes, the hemodynamics were similar to those of celiac axis lesions, and the rupture risk also might be considered high in this case.

There are reports of interventions for IPDA aneurysms, including endovascular or surgical techniques or a combination thereof.^2,6)^ The advantages of transcatheter arterial embolization (TAE) are its percutaneous and immediate performance without the need for general anesthesia. In this case, TAE was unfeasible because the aneurysm was near the SMA trunk, and TAE only may have induced severe ischemia of the liver, the pancreas, and the spleen. In patients for whom coil embolization is not feasible, covered stenting or stent-assisted coil embolization is another treatment option. However, it is limited by a suitable landing zone with a low degree of tortuosity.^2)^

The principal surgical procedures for IPDA aneurysms are ligation and resection or exclusion of aneurysms, with or without revascularization. Surgical bypass is necessary for revascularization. The advantage of a bypass is that it suppresses high blood flow through the peripancreatic arcades, thereby allowing control of newly forming aneurysms in these arcades and preventing organ ischemia.^7–9)^

In our case, hepatic and pancreatic perfusion mainly depended on the flow of peripancreatic arcades through the SMA. It is necessary to avoid the risk of hepatic ischemia, acute ischemic pancreatitis, and recurring aneurysms when selecting surgical techniques; therefore, we performed revascularization with aneurysmorrhaphy. We selected the iliac artery as an inflow of the bypass. It requires a long bypass route with an autologous vein or synthetic graft and is at risk of kinking and injuring the duodenum and ascending colon if a synthetic graft is being used. Still, it is easily exposed and bypassed under a favorable view. Organ and bowel ischemia risks are potentially much lower than with selecting a supra-celiac aorta or right renal artery as an inflow. However, the iliac artery is susceptible to occlusive disease over time; therefore, long-term follow-up is necessary to detect arterial and graft stenotic change and aneurysm recurrence, which may require prompt intervention.

## Conclusion

Hypoplasia of the celiac axis and its branches with an IPDA aneurysm is extremely rare. Normalization of blood flow with peripancreatic arcades is required to prevent organ ischemia and recurrent aneurysms. An ilio-hepatic artery bypass with an autologous vein graft is an effective treatment option.
